# ﻿*Geniostomaimadae* (Loganiaceae), a new single-island endemic species from Kaua‘i, Hawaiian Islands

**DOI:** 10.3897/phytokeys.257.154236

**Published:** 2025-06-10

**Authors:** Kenneth R. Wood, David H. Lorence, Warren L. Wagner, Susan Fawcett

**Affiliations:** 1 National Tropical Botanical Garden, 3530 Papalina Road, Kalāheo, HI 96741, USA National Tropical Botanical Garden Kalāheo United States of America; 2 Department of Botany, Smithsonian Institution, PO Box 37012, Washington, DC 20013-7012, USA Smithsonian Institution Washington United States of America; 3 University and Jepson Herbaria, 1001 Valley Life Sciences Building #2465, University of California, Berkeley, California 94720-2465, USA University of California Berkeley United States of America

**Keywords:** Conservation, discovery, endangered tree species, floristic diversity, Gentianales, *Geniostoma* sect. *Labordia*, Hawaiian flora, kāmakahala

## Abstract

A new endemic species of *Geniostoma* from Kaua‘i, Hawaiian Islands, is described and illustrated with notes on its distribution, ecology, conservation status, and relationships to other members of the genus. A modification to the existing key for Hawaiian *Geniostoma* is provided along with a key to all Kaua‘i species. *Geniostomaimadae***sp. nov.**, differs from its Hawaiian congeners by its unique combination of glabrous stems, leaves and stipules, flowers 3–10 in open, paniculate cymes with peduncles up to 75 mm long, corolla salverform, lobes 9–11 mm long, calyx lobes 5–9 mm long, and capsules 2-valved with beak 2–4 mm long. Population estimates range from 800 to 1250 individuals distributed across the central northern and eastern windward ridges, slopes and valleys of Kaua‘i. *Geniostomaimadae* represents a new Vulnerable (VU) single-island endemic species.

## ﻿Introduction

*Geniostoma* J.R.Forst. & G.Forst. (Loganiaceae) contains ca. 49 species of shrubs or small trees (rarely sprawling) distributed from Australasia to the Pacific, extending to the Mascarene Islands in the west, south through New Zealand, and north to southern Japan and the Hawaiian Islands (Gibbons et al. 2012). The Hawaiian lineage (known locally as kāmakahala) was previously placed in the endemic genus *Labordia* by Gaudichaud in 1829 (Gaudichaud-Beaupré 1829) and currently contains 17 species, one of which contains three varieties (Motley 1995; Wagner et al. 1999; Wood et al. 2007). Numerous botanists considered *Labordia* well distinguished from *Geniostoma*, by characters such as the terminal inflorescence (vs. axillary), elongated club-shaped stigma (vs. globose), larger flowers and capsules, corolla tubes much longer than the lobes, and capsules 2–3(4)-valved (vs. strictly 2-valved) (Gray 1859; Bentham and Hooker 1876; Hillebrand 1888; Wagner et al. 1999; Wood et al. 2007). However, Conn (1980) in his detailed revision of *Geniostoma* concluded that *Labordia* and *Geniostoma* were congeneric but recognized the Hawaiian species as belonging to Geniostomasubg.Labordia (Gaudich.) B.J.Conn, including two separate lineages, Geniostomasect.Labordia (Gaudich.) Baill. and G.sect.Darbolia Baill. Subsequent molecular phylogenetic studies by Gibbons et al. (2012) have confirmed that *Labordia* resolves with a clade of *Geniostoma* from the Pacific, which is sister to a clade of *Geniostoma* from New Guinea. Recent molecular phylogenetic analyses based on target-enrichment nuclear data using the *Angiosperms353* probes (Johnson et al. 2019; Lichter-Marck et al. unpubl. data) strongly support the monophyly of the two Hawaiian sections recognized by Conn (1980).

The island of Kaua‘i has the greatest diversity of *Geniostoma* in the Hawaiian Islands with eight species and two varieties, five of which are federally listed as endangered, and seven taxa being single island endemics. Ongoing studies on the Hawaiian lineage have brought to light a highly unique new member of Geniostomasect.Labordia, having open paniculate cymes on peduncles up to 75 mm long. We hereby describe and name this new Kaua‘i species *Geniostomaimadae* K.R.Wood, Lorence & W.L.Wagner, bringing the total number of Hawaiian *Geniostoma* to 20 taxa, and the number of single-island endemic vascular plant taxa on Kaua‘i to a very remarkable 262 (Rønsted et al. 2022; Wood and Knope 2023; Baldwin et al. 2024; Havran et al. 2024; Wood and Wagner 2024; Lorence et al. 2024; Wagner et al. 2024; Wood et al. 2024).

## ﻿Methods

Botanical voucher collections of *Geniostomaimadae* have been made over the past several decades and are curated at the PTBG herbarium with duplicates distributed to various herbaria, including BISH, F, CAS, MBK, MO, NY, P, and US (see Specimens examined). All morphological measurements were taken from dried herbarium specimens and field notes and are presented in the descriptions as follows: length × width, followed by units of measurements (mm, cm, or m). The authors have examined all 40+ specimens cited and have worked extensively with *Geniostoma* specimens at BISH, PTBG, and US. We assessed the extinction risk for *G.imadae* following the IUCN Red List Categories and Criteria (IUCN 2012, 2022). The extent of occurrence (EOO) and area of occupancy (AOO) were calculated by using ArcMap 10.6.1 in relation to coordinates recorded while collecting herbarium specimens or making field observations. Geographic coordinates have been truncated to protect exact locations from unauthorized access.

## ﻿Taxonomic treatment

### ﻿ Geniostoma imadae

Taxon classificationPlantaeGentianalesLoganiaceae

K.R.Wood, Lorence & W.L.Wagner sp. nov.

350BA544-F257-5C63-809A-4BE99B4BF1DD

urn:lsid:ipni.org:names:77363123-1

Figs 1 , 2 , 3A, B

#### Diagnosis.

*Geniostomaimadae* is morphologically most similar to *G.degeneri* (Sherff) Byng & Christenh. from which it differs by its combination of leaves abaxially glabrous (vs. hirtellous), peduncle length (15–)20–75 mm long (vs. sessile), and capsule length 10–15 mm long with no keel (vs. 20–30 mm long with keel).

#### Type.

**USA. Hawaiian Islands, Kaua‘i**: Līhu‘e District, ‘Iole headwaters, ♀, 22.042, -159.497, 902 m alt., 29 Jul 2021 (fr.), *K.R. Wood, S. Heintzman & S. Deans 18793* (holotype: PTBG1000098358!; isotypes (to be distributed): BISH!, CAS!, US!).

#### Description.

***Shrubs or small trees***, 1–3.5 m tall; trunk 2–5 cm diameter near base, bark gray to gray-brown; stems terete, lateral branches dichotomously branched, young and old stems glabrous. ***Leaves*** opposite, pinnately nerved, upper surface medium green, lower surface pale green; blade 3–12(–14) × 1.2–4.5(–5.5) cm, coriaceous, elliptic to elliptic-obovate, young and mature leaves glabrous on upper and lower surface, margins entire, apex apiculate to obtuse, base cuneate to acuminate, petioles (1–)2–15(–18) mm long; stipules interpetiolar, glabrous, completely connate, forming a truncate sheath 4–8(–11) mm long, usually splitting with age, adnate to the petiole at base. ***Flowers*** functionally unisexual, plants dioecious, inflorescence in pendulous, open paniculate cymes, flowers 3–8 in pistillate individuals, 3–10 in staminate plants, peduncles glabrous, recurved, (15–)20–40 mm long, elongating to 75 mm in fruit, pedicels glabrous, 10–40 mm long, elongating to 50 mm in fruit, bracts and bracteoles linear subulate, 2–5.5 × 0.1–0.8(–2.5) mm; calyx lobes 5(–6), connate near base, imbricate, linear-lanceolate or ovate-lanceolate, glabrous, apex acuminate, 5–9 × 0.8–3 mm, 5–7 nerved, margin hyaline; corolla salverform, 5(–6)-lobed, orange or yellow, fleshy, glabrous externally, inner tube pilose below throat, staminate and pistillate flowers 12–22 mm long, the tube 8–12 × 1–4(–5.5) mm, sparsely pilose within becoming densely pilose near throat, the lobes reflexed at anthesis, linear, 9–11 mm long, apex acuminate to acute; anthers 5(–6), adnate to corolla tube, dorsifixed, slightly exerted; staminate flowers with anthers 2.1–4 × 0.8–1.2 mm, reduced ovary glabrous 4.0 × 0.9 mm, pistil 12.5–18 mm long, stigma cylindrical and branched 5–14 mm long, style 2–4 mm long; pistillate flowers with anthers reduced, 1.2–1.8 × 0.4–0.8 mm, ovary glabrous 5.0 × 2.5 mm, pistil 12–12.5 mm long, stigma cylindrical and branched 5 mm long, style 2 mm long. ***Capsules*** green, light brown at maturity, ovoid-ellipsoid, apex acuminate, 10–15 mm long, 2-valved, valves transversely wrinkled, not keeled, apex with beak, 2–4 mm long. ***Seeds*** ovoid-ellipsoid, brown, 1.2–1.5 × 0.7–0.8 mm, embedded in orange pulp.

**Figure 1.**
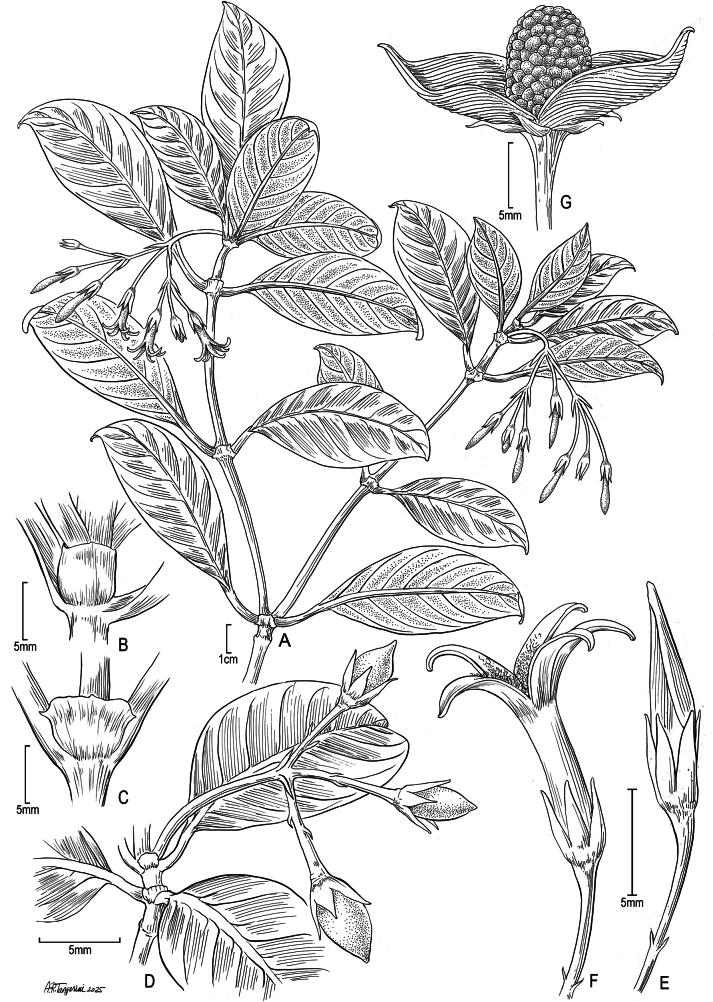
*Geniostomaimadae* K.R.Wood, Lorence & W.L.Wagner **A** flowering branch **B** young connate interpetiolar stipule forming truncate sheath **C** stipule splitting with age **D** fruiting branch **E** unopened flower, lateral view with imbricate lobes **F** mature open flower, lateral view **G** open 2-valved capsule with transverse ridges and seeds embedded in pulp. Drawn from: **A***Perlman 18532* (PTBG, US), *Wood & Query 12819* (PTBG, US), *Wood 15474* (PTBG) **B** field photo taken 20 Nov 2024 **C***Wood, Perlman & Query 15930* (PTBG, US), *Perlman & Hill 17929* (PTBG, US) **D***Wood, Heintzman & Deans 19365* (PTBG) **E, F***Perlman & Hill 17929* (PTBG, US) **G***Perlman & Kishida 22954* (PTBG, US) (Illustration by Alice Tangerini).

#### Additional specimens examined (paratypes).

**USA. Hawaiian Islands, Kaua‘i: Hanalei**; • 1 ♀; 983 m alt.; 23 May 2022; *Williams AMW733* (PTBG) • ‘**Ili‘ili‘ula**; 1 ♀; 1234 m alt.; 3 Apr 2013; *Wood 15474* (BISH, PTBG) • 1 ♀; *loc. cit.*; 792 m alt.; 30 Jun 2021; *Wood et al. 18758* (PTBG) • ‘**Iole**; 1 ♀; 927 m alt.; 10 Jan 2012; *Wood 14834* (PTBG) • 1 ♀; *loc. cit.*; 774 m alt.; 28 Aug 2013; *Wood et al. 15655* (PTBG) • 1 ♀; *loc. cit.*; 905 m alt.; 10 Aug 2023; *Wood et al. 19365* (BISH, PTBG, US) • 1 ♀; *loc. cit.*; 890 m alt.; 7 Aug 2024; *Wood et al. 19587* (BISH, CAS, MO, NY, PTBG, US) • 1 ♀; *loc. cit.*; 914 m alt.; 3 Oct 2024; *Wood & Heintzman 19638* (PTBG) • **Kamo‘oloa**; 1 ♀; 914 m alt.; 4 Oct 1996; *Wood 5686* (PTBG, US) • 1 ♂, *loc. cit.*; 905 m alt.; 21 Feb 2008; *Wood & Query 12802* (BISH, PTBG) • 1 ♂; *loc. cit.*; 884 m alt.; 21 Feb 2008; *Wood & Query 12819* (BISH, NY, PTBG, US) • 1 ♀; *loc. cit.*; 923 m alt.; 27 Aug 2015; *Walsh et al. SKW90* (BISH, PTBG) • **Lumaha‘i**; 1 ♀; 792 m alt.; 23 Jul 2024; *Wood et al. 19570* (BISH, PTBG, US) • **Wahiawa**; 1 ♂; 823 m alt.; 9 May 1972; *Herbst & Takahashi 2401* (BISH, PTBG) • 1 ♂; *loc. cit.*;716 m alt.; 3 Mar 1987; *Flynn 2067* (PTBG) • 1 ♀; *loc. cit.*;700 m alt.; 7 Feb 1991; *Flynn et al. 4416* (PTBG) • 1 ♀; *loc. cit.*; 720 m alt.; 7 Feb 1991; *Wood et al. 0576* (PTBG) • 1 ♂; *loc. cit.*; 700 m alt.; 28 Mar 1991; *Wood et al. 0690* (PTBG) • 1 ♀; *loc. cit.*; 700 m alt.; 28 Mar 1991; *Lorence et al. 6741* (BISH, F, MO, PTBG, US) • 1 ♀; *loc. cit.*; 790 m alt.; 10 Apr 1991; *Flynn et al. 4615* (BISH, PTBG, US) • 1 ♀; *loc. cit.*; 13 Apr 1991; *Flynn et al. 4591* (PTBG) • 1 ♀, *loc. cit.*; 770 m alt.; 20 Apr 1991; *Flynn et al. 4611* (BISH, PTBG, US) • 1 ♀; *loc. cit.*; 870 m alt.; 13 May 1991; *Lorence et al. 6785* (PTBG) • 1 ♀, *loc. cit.*; 930 m alt.; 15 May 1991; *Wood et al. 0840-A* (PTBG) • 1 ♀; *loc. cit.*; 825 m alt.; 20 May 1991; *Wood et al. 0865* (PTBG) • 1 ♂; *loc. cit.*; 600 m alt.; 1 Jul 1991; *Wood et al. 0997* (PTBG, US) • 1 ♂; *loc. cit.*; 762 m alt.; 12 May 1995; *Wood 4272* (PTBG) • 1 ♀; *loc. cit.*; 762 m alt.; 12 May 1995; *Wood 4273* (NY, PTBG) • 1 ♀; *loc. cit.*; 732 m alt.; 15 Jan 2002; *Perlman & Hill 17891* (BISH, NY, PTBG, US) • 1 ♂; *loc. cit.*; 975 m alt.; 28 Feb 2002; *Perlman & Hill 17920* (BISH, MO, NY, PTBG) • 1 ♀; *loc. cit.*; 914 m alt.; 28 Feb 2002; *Perlman & Hill 17929* (PTBG, US) • 1 ♂; *loc. cit.*; 899 m alt.; 4 Apr 2003; *Perlman 18532* (MO, PTBG, US) • 1 ♀; *loc. cit.*; 797 m alt.; 6 Apr 2009; *Tangalin et al. 2003* (MBK, PTBG, US) • 1 ♀; *loc. cit.*; 783 m alt.; 28 Jun 2012; *Perlman & Kishida 22954* (BISH, NY, PTBG, US) • 1 ♀; *loc. cit.*; 725 m alt.; 2 Nov 2012; *Perlman 23089* (PTBG) • 1 ♀; *loc. cit.*; 759 m alt.; 23 May 2013; *Perlman & Kishida 23474* (PTBG) • **Waiahi**; 1 ♀; 914 m alt.; 25 Nov 2013; *Wood et al. 15743* (BISH, PTBG, US) • 1 ♀; *loc. cit.*; 792 m alt.; 30 Dec 2013; *Wood et al. 15770* (BISH, PTBG, US) • 1 ♂; *loc. cit.*; 790 m alt.; 4 Apr 2019; *Wood et al. 18141* (BISH, NY, PTBG, US) • 1 ♂; *loc. cit.*; 805 m alt.; 4 Apr 2019; *Wood et al. 18144* (PTBG, US) •1 ♀; *loc. cit.*; 815 m alt.; 4 Apr 2019; *Wood et al. 18154* (PTBG, US) • **Wainiha**; 1 ♀; 732 m alt.; 23 Apr 2014; *Wood et al. 15930* (BISH, CAS, PTBG, US).

**Figure 2.**
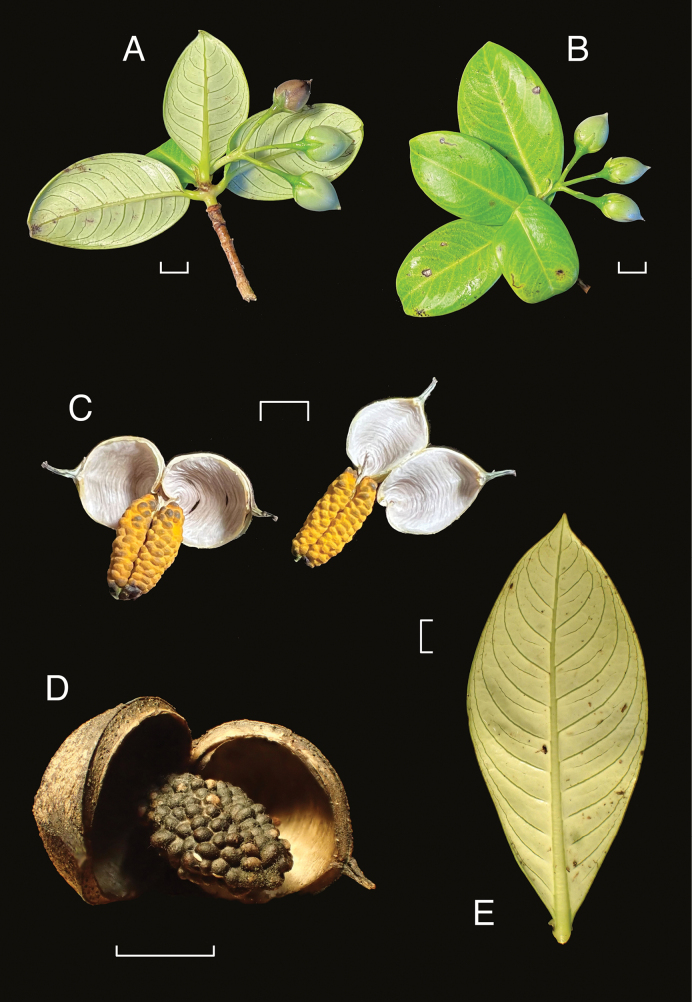
*Geniostomaimadae* K.R.Wood, Lorence & W.L.Wagner **A, B** fruiting branches, unopened capsules with abaxial and adaxial leaf surfaces **C** freshly opened capsules showing apex with long beak, valves transversely wrinkled and seeds embedded in orange pulp **D** mature, open capsule with seeds embedded in dried pulp **E** abaxial surface of large leaf. Field photos: **A, B***Wood, Heintzman & Deans 19365* (BISH, PTBG, US) **C***Wood & Heintzman 19638* (PTBG) **D***Wood, Heintzman & Deans 19587* (BISH, CAS, MO, NY, PTBG, US) **E***Wood, Heintzman & Deans 19570* (BISH, PTBG, US). Scale bars: 1 cm (**A, B, E**); 5 mm (**C, D**). Photos by K.R. Wood.

**Figure 3.**
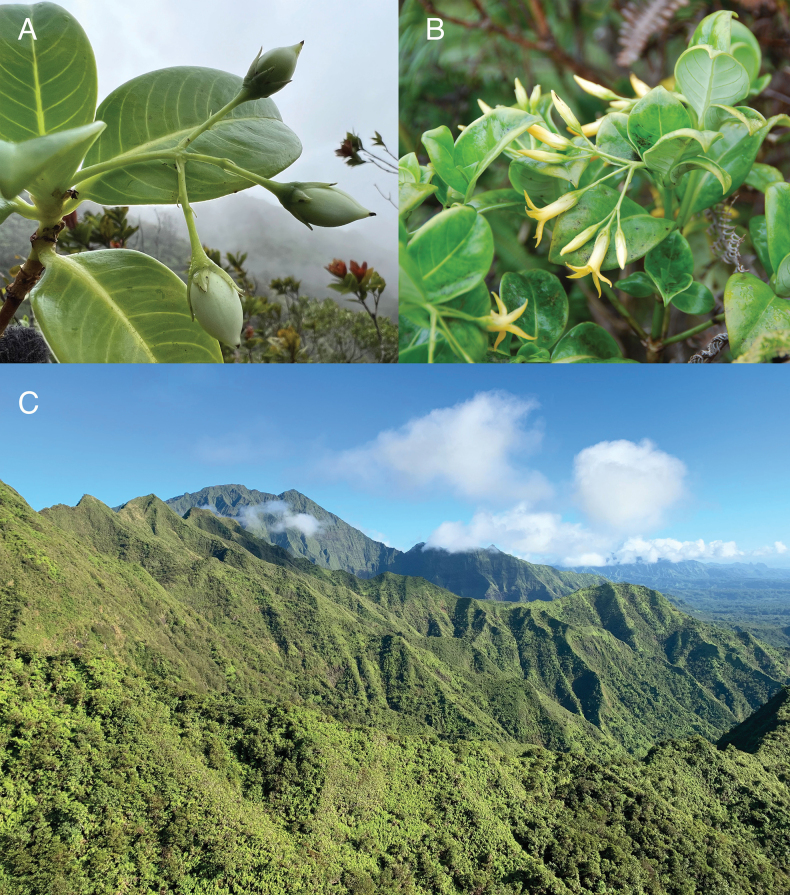
**A, B***Geniostomaimadae* K.R.Wood, Lorence & W.L.Wagner. **A** Fruiting branch *in situ***B** flowering branch *in situ***C** habitat, looking north along the main windward ridge of Kaua‘i, toward the Kawaikini summit. Field photos: **A***Wood, Heintzman & Deans 19365* (BISH, PTBG, US) **B***Wood 15474* (BISH, PTBG) **C** photo taken 14 Nov 2019. Photos by K.R. Wood.

#### Phenology.

*Geniostomaimadae* has been observed with flower during the months of January to August, and with fruit October to February and April to August.

#### Etymology.

*Geniostoma* is derived from the Greek *geneion*, beard, and *stoma*, mouth, referring to the hairs on the inner rim of the corolla (Smith and Stone 1962; Lorence and Wagner 2020). The epithet of this new species recognizes Clyde Imada, Research Specialist at the Bishop Museum. It is with great respect and admiration for his many contributions to Hawaiian botany that we name this species in his honor.

#### Vernacular name.

Kāmakahala is the Hawaiian name for related species. The highly prized flowers of the Hawaiian members of the genus were used for leis and wreaths and reserved only for high chiefs (Hillebrand 1888).

#### Affinities.

Molecular phylogenetic analyses based on target-enrichment nuclear data using the *Angiosperms353* probe set (Johnson et al. 2019; Lichter-Marck et al. unpubl. data) support the recognition of two distinct lineages in the Hawaiian Islands, corresponding to Geniostomasubg.Labordiasect.Labordia and G.sect.Darbolia recognized by Conn (1980). The new taxon belongs to sect. Labordia, which is characterized by corollas orange-yellow and usually salverform in shape (vs. sect. Darbolia with corollas white to greenish and urceolate) (Table 1).

**Table 1.** Comparison of morphological characters for all nine species of *Geniostoma* on Kaua`i.

	*G.imadae*	*G.degeneri*	*G.helleri*	*G.hirtellum*	*G.lorencianum*	*G.lydgatei*	*G.pumilum*	*G.tinifolium*	*G.waialealae*
**Pubescence on young stems**	glabrous	hispidulous or glabrate	glabrous	hirtellous	tomentose	hispidulous	glabrate	glabrous	hispid
**Young stems angled, winged, or terete**	terete	terete or weakly angled	terete	sharply angled to winged	terete	sharply angled	sharply angled to winged	terete	angled
**Pubescence on abaxial leaf surface**	glabrous	hirtellous	glabrous	hispid	tomentose	hispidulous	glabrous	glabrous	hispidulous
**Leaf base**	cuneate to acuminate	subtruncate to cuneate	cuneate	cuneate	cordate to auriculate	attenuate To cuneate	cuneate	cuneate	cuneate
**Stipule length**	4–8(–11) mm	6–11 mm	1–5 mm	2–6(–8) mm	1.5–2 mm	4–5 mm	5–9 mm	1–4 mm	1.8–2.5 mm
**Stipule pubescence**	glabrous	glabrous	glabrous	glabrous	hirsute to tomentose	glabrous or hispidulous	glabrous	margins ± ciliate	hispidulous
**Peduncle length**	(15–)20–75 mm	sessile	5–20 mm	sessile	20–60 mm	sessile	0–6 mm	9–35 mm	subsessile
**Flower** #	3–10	3–10(–15)	3–9	(3–)6–25(–50)	3–34	6–20	1–15	(3–)9–12(–19)	3–12
**Corolla shape & color**	salverform, orange-yellow	salverform, orange-yellow	urceolate, white or pale green-yellow	salverform, green-yellow or yellow-orange	urceolate, green	salverform, pale yellow	salverform, orange	urceolate, green-yellow	salverform, yellow
**Corolla tube length**	8–12 mm	10–11 mm	7–8 mm	(11–)14– 23(–28) mm	10–13 mm	4 mm	9–10 mm	5.5–7.8 mm	5.5–10 mm
**Corolla lobe length**	9–11 mm	8–10 mm	2–3.5 mm	7–15(–18) mm	1.4–1.7 mm	2.5–3 mm	5–7 mm	1.7–2.3 mm	3–5 mm
**Calyx lobe length**	5–9 mm	6–15 mm	1.7–2.5 mm	3–10 mm	2.0–2.7 mm	1.5–2(–2.5) mm	6–11 mm	1.5–3 mm	3–6.5 mm
**Calyx lobe width**	0.8–3 mm	3–4 mm	0.8–1.3 mm	0.8–2(–2.5) mm	0.9–1.3 mm	0.5–0.8 mm	2–3 mm	0.7–1.6 mm	0.4–1.2 mm
**Capsule valves**	transversely wrinkled	transversely wrinkled	longitudinally striate	transversely wrinkled	longitudinally striate	transversely wrinkled	transversely wrinkled	transversely wrinkled	transversely wrinkled
**Capsule length**	10–15 mm	20–30 mm	9–20 mm	15–34 mm	25–37 mm	3.5–4 mm	9–14 mm	8–17 mm	9–15 mm
**Capsule w/ keel**	no	yes	no	no	no	no	yes	yes	yes
**Capsule valves**	2-valved	2-valved	2(3)-valved	2(3)-valved	2-valved	2-valved	3-valved	2(3)-valved	2(3)-valved
**Capsule beak length**	2–4 mm	1–2.5 mm	0.7–1.2 mm	(1.5–)2.5–6 mm	3.5 mm	0.2–0.3 mm	1.5–2.5 mm	0.5–1.5 mm	0.2–1.8 mm

**Figure 4.**
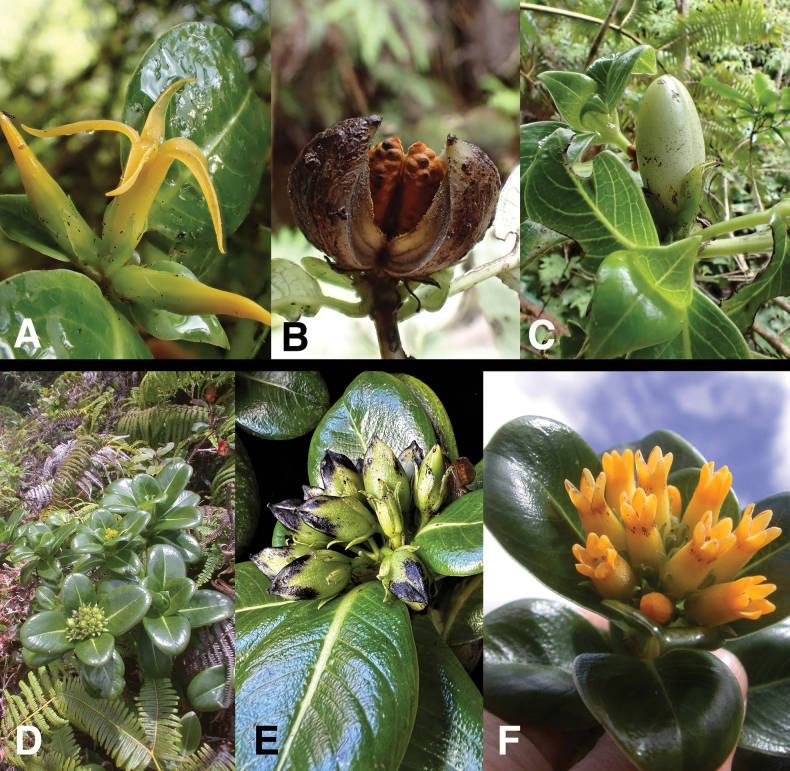
**A–C***Geniostomadegeneri* (Sherff) Byng & Christenh. **A** Flowers on sessile cyme **B** mature keeled capsule with seeds embedded in orange pulp **C** immature fruit **D–F***Geniostomapumilum* (Hillebr.) Byng & Christenh. **D** habit with flowers and fruit on sessile cymes **E** fruiting branch with keeled 3-valved capsules **F** flowers with short lobes on sessile cyme. Field photos: **A***Wood 17342* (BISH, NY, PTBG, UC, US) **B***Wood et al. 17514* (PTBG) **C***Wood & Query 18022* (BISH, PTBG) **D***Wood 12291* (BISH, PTBG) **E***Wood, Heintzman & Deans 19272* (BISH, CAS, PTBG, US) **F***Wood 12304* (PTBG). Photos by K.R. Wood.

Morphologically, *Geniostomaimadae* is most similar to *G.degeneri* (Fig. 4A–C), yet can be easily separated by features stated in the diagnosis. The leaves, stipules and flowers of *G.imadae* are also quite similar to *G.pumilum* (Hillebr.) Byng & Christenh. (Fig. 4D–F), yet *G.imadae* starkly differs in having stems terete (vs. sharply angled or winged), peduncles 20–75 mm long (vs. 0–6 mm), corolla lobes 9–11 mm long (vs. 5–7 mm), corolla glabrous externally (vs. hirsute), and capsules not keeled, 2-valved (vs. keeled, 3-valved). Interestingly, *G.imadae* has been misidentified as *G.tinifolium* (A.Gray) B.J.Conn because of their superficial similarity in having long pedunculate cymes, yet both belong to different lineages with *G.imadae* being a member of sect. Labordia (vs. sect. Darbolia for *G.tinifolium*). Peduncle length has been used to distinguish these sections previously, but is not congruent with floral traits and molecular phylogenetic evidence. Taxa with elongate peduncles are compared in Table 2. *Geniostomaimadae* clearly differs from *G.tinifolium* in having corolla tubes 8–12 mm long (vs. 5.5–8 mm), corolla lobes 9–11 mm long (vs. 1.5–3 mm), corolla color orange-yellow (vs. green-yellow), corolla shape salverform (vs. urceolate) stipules glabrous (vs. ± ciliate), and capsules not keeled, 2-valved (vs. keeled, 2(3)-valved). (Tables 1, 2).

**Table 2.** Comparison of morphological characters for species of Hawaiian *Geniostoma* having peduncles >7 mm long.

	Geniostomasubg.Labordiasect.Labordia		Geniostomasubg.Labordiasect.Darbolia				
**Species**	***G.imadae***	***G.cyrtandrae***	***G.helleri***	***G.kaalae***	***G.lorencianum***	***G.tinifolium***	***G.triflorum***
**Pubescence on stems**	glabrous	short-hispidulous	glabrous	short-hispidulous	tomentose	glabrous	glabrous
**Pubescence on lower leaves**	glabrous	short-hispidulous	glabrous	short-hispidulous	tomentose	glabrous	glabrous
**Leaf base**	cuneate to acuminate	cuneate	cuneate	cordate to truncate	cordate to auriculate	cuneate	cordate
**Stipule length**	4–8(–11) mm	3–7 mm	1–5 mm	1.5–2.4 mm	1.5–2 mm	1–4 mm	1–4 mm
**Stipule pubescence**	glabrous	margins ± ciliate	glabrous	margins ± ciliate	hirsute to tomentose	margins ± ciliate	margins ± ciliate
**Peduncle length**	20–75 mm	0–10 mm	5–20 mm	15–50 mm	20–60 mm	9–35 mm	40–80 mm
**Capsule valves**	transversely wrinkled	transversely wrinkled	longitudinally striate	transversely wrinkled	longitudinally striate	transversely wrinkled	transversely wrinkled
**Corolla tube length**	8–12 mm	15–22 mm	7–8 mm	9–10 mm	10–13 mm	5.5–7.8 mm	6–10 mm
**Corolla lobe length**	9–11 mm	8–13 mm	2–3.5 mm	2–3 mm	1.4–1.7 mm	1.7–2.3 mm	1.5–2.5 mm
**Corolla color**	orange-yellow	pale yellow	white or green-yellow	green or green-yellow	green	green-yellow	green-yellow
**Corolla shape**	salverform	salverform	urceolate	urceolate	urceolate	urceolate	urceolate

#### Distribution and ecology.

**Figure 5.**
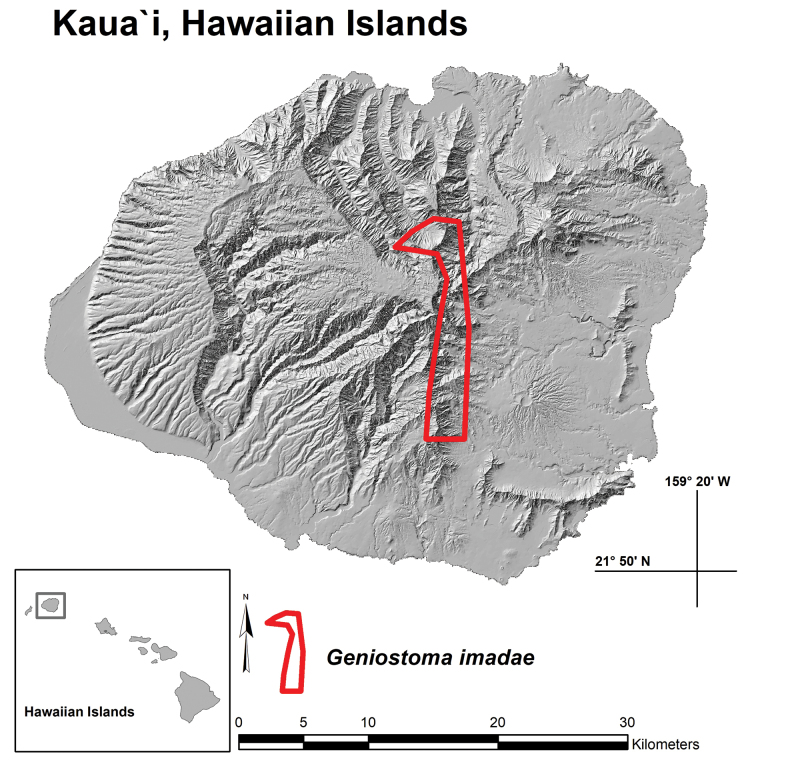
Distribution map (Kaua‘i, Hawaiian Islands) with polygon indicating known range of *Geniostomaimadae* K.R.Wood, Lorence & W.L.Wagner.

*Geniostomaimadae* is endemic to the volcanic island of Kaua‘i, where it is has been documented along the central northern ridges, slopes and riparian valleys of Wainiha, Lumaha‘i and Hanalei and extends south along the islands central eastern windward regions of ‘Ili‘ili‘ula, ‘Iole, Kamo‘oloa, Waiahi, and Wahiawa (Figs 3C, 5). We estimate ca. 800 to 1250 individuals occur in lowland to montane wet forests ranging in elevation between 600–1234 m and dominated by trees of *Metrosideros* Banks ex Gaertn. (Myrtaceae) and *Cheirodendron* Nutt. ex Seem. (Araliaceae). Greater numbers of *G.imadae* are found along forested stream banks but also inhabit upper forested slopes that extend up to dividing ridges and border expansive patches of matting ferns such as *Dicranopteris* Bernh. and *Diplopterygium* (Diels) Nakai (Gleicheniaceae). Associated genera of trees and shrubs include *Polyscias* J.R.Forst. & G.Forst. (Araliaceae); *Pritchardia* Seem. & H.Wendl. (Arecaceae); *Dubautia* Gaudich. (Asteraceae); *Cyanea* Gaudich. (Campanulaceae), *Perrottetia* Kunth (Dipentodontaceae); *Antidesma* L., *Euphorbia* L. (Euphorbiaceae); *Hydrangea* Gronov. (Hydrangeaceae); *Geniostoma* (Loganiaceae); *Myrsine* L. (Primulaceae); *Syzygium* Gaertn. (Myrtaceae); *Bobea* Gaudich., *Coprosma* J.R.Forst. & G.Forst., *Kadua* Cham. & Schltdl., *Psychotria* L. (Rubiaceae); *Melicope* J.R.Forst. & G.Forst. (Rutaceae); and *Pipturus* Wedd., *Touchardia* Gaudich. (Urticaceae). Genera of sedges and grasses include *Carex* L., *Cyperus* L., *Machaerina* Vahl (Cyperaceae); *Eragrostis* Wolf, *Panicum* L. (Poaceae); herbs and sub-shrubs include *Bidens* L. (Asteraceae); *Vaccinium* L. (Ericaceae); *Cyrtandra* J.R.Forst. & G.Forst. (Gesneriaceae); and *Freycinetia* Gaudich. (Pandanaceae). Genera of ferns include *Asplenium* L., *Hymenasplenium* Hayata (Aspleniaceae); *Deparia* Hook. & Grev., *Diplazium* Sw. (Athyriaceae); *Sadleria* Kaulf. (Blechnaceae); *Cibotium* Kaulf. (Cibotiaceae); *Microlepia* C.Presl (Dennstaedtiaceae); *Ctenitis* (C.Chr.) C.Chr., *Dryopteris* Adans. (Dryopteridaceae); *Hoiokula* S.E.Fawc. & A.R.Sm., and *Menisciopsis* (Holttum) S.E.Fawc. & A.R.Sm. (Thelypteridaceae). There is also a diverse association of terrestrial and epiphytic lichens and bryophytes. The holotype of *Geniostomaimadae* was collected in the immediate area of the recently discovered and described species *Melicopeiolensis* K.R.Wood, Lorence & W.L.Wagner (i.e., ‘Iole Valley, Wood et al. 2024), attesting to the floristic diversity of the region and the importance of ongoing botanical inventories and conservation.

##### ﻿Key to Hawaiian *Geniostoma*

The following key to Hawaiian *Geniostoma* (treated as *Labordia* by Wagner, Herbst, and Sohmer in Wagner et al. 1990, pp. 853–854) has been modified in couplets 1–7 to accommodate *Geniostomaimadae* sp. nov.

Note: *Geniostomalorencianum* (K.R.Wood, W.L.Wagner & T.Motley) Byng & Christenh. and *G.triflorum* (Hillebr.) Byng & Christenh., which were described or resurrected respectively post Wagner et al. 1990, are also included in this modified key. HI = Hawaiian Islands; H = Hawai‘i (Big Island); K = Kaua‘i; Ka = Kaho‘olawe; L = Lana‘i; M = Maui; Mo = Moloka‘i; Ni = Ni‘ihau; O = O‘ahu.

**Table:** 

1	Cymes on a common peduncle (7–)9–40 mm long, elongating up to 80 mm in fruit; capsule valves not or obscurely keeled	**2**
–	Cymes sessile or on a common peduncle 1–5(–6) mm long; capsule valves keeled or in *G.hirtellum* and *G.lydgatei* without a keel	**8**
2(1)	Young stems and lower leaf surface densely tomentose, leaf base cordate to auriculate; K	***G.lorencianum***
–	Young stems and lower leaf surface not tomentose, leaf base not auriculate	**3**
3(2)	Young stems and lower leaf surface short-hispidulous, sometimes sparsely so	**4**
–	Young stems and lower leaf surface glabrous	**5**
4(3)	Leaves (12–)15–30 cm long; peduncles 3–10 mm long; corolla tube 15–22 mm long; capsules lanceoloid-ellipsoid, 32–35 mm long; O	***G.cyrtandrae* Baill.**
–	Leaves 6–17 cm long; peduncles 15–50 mm long; corolla tube 9–10 mm long; capsules broadly ovoid, 12–16 mm long; O (Wai‘anae Mts.)	***G.kaalae* (C.N.Forbes) K.L.Gibbons, B.J.Conn & M.J.Henwood**
5(3)	Capsule valves longitudinally striate; stipules glabrous; corolla lobes 2–3.5 mm long; K	***G.helleri* (Sherff) Byng & Christenh.**
–	Capsule valves transversely wrinkled; stipules glabrous, with margins ± ciliate except in *G.imadae*	**6**
6(5)	Corolla green-yellow, urceolate, lobes 1.5–2.5 mm long	**7**
–	Corolla orange-yellow, salverform, lobes 9–11 mm long; K	***G.imadae***
7(6)	Leaf petioles 1–3 mm long, leaf base cordate, blade lanceolate to elliptic-lanceolate; peduncles 40–50 mm long, elongating to 70–80 mm in fruit; pedicels 10–25 mm long, elongating to 30 mm at maturity; plants often scandent in habit; SE Moloka‘i	***G.triflorum***
–	Leaf petioles 6–22(–40) mm long, leaf base cuneate, blade elliptic to elliptic-oblanceolate; peduncles 9–22 mm long, elongating to 13–25(–35) mm in fruit; pedicels 8–11 mm long, elongating to 23 mm at maturity; plants tree-like in habit; HI except Ni & Ka.	***G.tinifolium***
8(1)	Calyx lobes large and foliaceous, rhombic-oblanceolate, rhombic-obovate, rhombic, or sometimes suborbicular, 15–31 mm long, the outer ones 6–12 mm wide, enclosing capsules; flowers usually solitary, rarely up to 11 per cyme; O, Mo, L	***G.waiolani* (Wawra) Byng & Christenh.**
–	Calyx lobes not foliaceous, variously shaped, but not rhombic, 1.5–16 mm long, 0.4–7 mm wide, not entirely enclosing capsules; flowers (2–)3–80 per cyme	**9**
9(8)	Calyx lobes 1.5–2(–2.5) mm long; capsules 3.5–4 mm long, with a blunt beak 0.2–0.3 mm long; K	***G.lydgatei* (C.N.Forbes) Byng & Christenh.**
–	Calyx lobes 3–16 mm long; capsules 9–35 mm long, with a beak 1–7 mm long, except in *G.waialealae* with beak 0.2–1.8 mm long	**10**
10(9)	Stipules 1.8–2.5 mm long, short-hispidulous at least at base; leaves concave when fresh, margins irregularly revolute when pressed; K	***G.waialealae* (Wawra) Byng & Christenh.**
–	Stipules 3–14 mm long or, if as short as 2 mm long, then glabrous; leaves flat or concave when fresh, margins flat to revolute but not irregularly so when pressed	**11**
11(10)	Calyx lobes broadest above middle, spatulate to oblanceolate	**12**
–	Calyx lobes broadest below or at middle, linear-lanceolate, lanceolate, oblong-lanceolate, or sometimes narrowly elliptic	**13**
12(11)	Upper leaf surface with veins slightly impressed, lower surface glabrous or sparsely short-hispid, sometimes only along veins; stipules adnate in lower 1/3 to petioles; capsules 3-valved; pedicels glabrous or sparsely to rarely moderately short-hispidulous; O (Ko‘olau Mts)	***G.hymenopodum* (O.Deg. & Sherff) Byng & Christenh.**
–	Upper leaf surface with veins impressed, lower surface appressed short-hispid along the conspicuously raised veins; stipules weakly adnate to petioles only at base; capsules 2(3)-valved; pedicels densely short-hispidulous, more sparsely so in fruit; East Maui	***G.venosum* (Sherff) Byng & Christenh.**
13(11)	Leaves 1.5–5(–7) cm long, strongly rugose; low-growing, many-branched shrubs up to ca. 0.5 m tall; O (Ko‘olau Mts.)	***G* . *hosakanum* (Sherff) Byng & Christenh.**
–	Leaves (3.5–)4–30 cm long, flat or veins slightly impressed; erect or sometimes scandent shrubs or small trees 0.4–6 m tall	**14**
14(13)	Calyx lobes linear-lanceolate or linear-subulate, 0.8–1.8(–2.5) mm wide; corolla pale orange, yellow, greenish yellow, or cream, the tube narrowly urceolate, (1.8–)2 or more times longer than calyx; capsules not or obscurely keeled	**15**
–	Calyx lobes oblong-lanceolate to lanceolate-ovate or narrowly elliptic, sometimes linear-lanceolate, 1.8–6 mm wide; corolla yellow to orange, the tube salverform-funnelform, as long as or up to 1.5 times as long as calyx; capsules usually keeled	**16**
15(14)	Stems fleshy, terete or weakly angled, becoming flattened when dry, short-hispidulous; bracts filiform, 3–6 mm long; corolla tube 15–22 mm long, the lobes 8–13 mm long; O	***G.cyrtandrae***
–	Stems not or slightly fleshy, usually sharply angled or winged and short-hirtellous, if not angled or winged, then glabrous; bracts linear-spatulate to filiform, 8–10 mm long; corolla tube 7–15(–18) mm long, the lobes 4–8.5(–10) mm long; HI exc. Ni & Ka	***G.hirtellum* (H.Mann) Byng & Christenh.**
16(14)	Stems terete or obscurely angled; capsules 2–30 mm long, slightly fleshy and white at maturity; corolla lobes 8–10 mm long; K	***G.degeneri***
–	Stems angled or slightly winged; capsules 9–20 mm long, not fleshy and green turning brown at maturity; corolla lobes 3.5–12 mm long	**17**
17(16)	Bracts and bracteoles 3.5–4.5 mm long; stipules 3–4 mm long, adnate in lower 1/3–1/2 to petioles; calyx lobes inconspicuously nerved; leaves 5–8 cm long, 2–3 cm wide; O (Ko‘olau Mts)	***G.gaudichaudii* B.J. Conn**
–	Bracts 4.8–9 mm long, bracteoles 4–9 mm long; stipules 3–9 mm long, adnate to petioles only at base or distinct; calyx lobes conspicuously 5–7-nerved or only midvein conspicuous; leaves (3.5–)4–15 cm long, 1.8–6 cm wide	**18**
18(17)	Lower leaf surface with veins conspicuously raised, appressed short-hispid along veins, sometimes with hairs scattered over surface; corolla tube 4.5–8 mm long; East Maui	***G.venosum***
–	Lower leaf surface with veins not or only slightly raised, glabrous or sparsely to sometimes moderately short-hispid, especially or exclusively on veins; corolla tube 6.8–15 mm long	**19**
19(18)	Stipules 3–6 mm long; bracts and bracteoles 6–9 mm long; capsules 12–20 mm long, 2–3(4)-valved, the valves with a keel 1.5–3.5 mm wide and apex with a beak 3.5–7 mm long; lower leaf surface ± short-hispid; Mo, L, M, H	***G.hedyosmifolium* (Baill.) Byng & Christenh.**
–	Stipules 5–9 mm long; bracts and bracteoles 4–7 mm long; capsules 9–14 mm long, 3-valved, the valves with a keel 0.5–1.5 mm wide and apex with a beak 1.5–2.5 mm long; lower leaf surface glabrous; K	***G.pumilum***

##### ﻿Key to Kaua`i *Geniostoma*

**Table:** 

1	Abaxial leaf surface glabrous	**2**
–	Abaxial leaf surface hirtellous, hispid, hispidulous, or tomentose	**5**
2(1)	Capsule valves longitudinally striate	***G.helleri***
–	Capsule valves transversely wrinkled	**3**
3(2)	Young stems sharply angled to winged	***G.pumilum***
–	Young stems terete	**4**
4(3)	Stipules glabrous, 4–8(–11) mm long; corolla tubes 8–12 mm long, corolla lobes 9–11 mm long; calyx lobes 5–9 mm long; capsules without keel, 2-valved with beak 2–4 mm long	***G.imadae***
–	Stipules with margins ± ciliate, 1–4 mm long; corolla tubes 5.5–7.8 mm long, corolla lobes 1.7–2.3 mm long; calyx lobes 1.5–3 mm long; capsules with keel, 2(3)-valved with beak 0.5–1.5 mm long	***G.tinifolium***
5(1)	Abaxial leaf surface tomentose, leaf base cordate to auriculate; peduncles 20–60 mm long; capsule valves longitudinally striate	***G.lorencianum***
–	Abaxial leaf surface hirtellous, hispid, or hispidulous, leaf base cuneate to attenuate or sub-truncate; inflorescences sessile to sub-sessile; capsule valves transversely wrinkled	**6**
6(5)	Corolla tubes 4 mm long; capsules 3.5–4 mm long	***G.lydgatei***
–	Corolla tubes 5.5–28 mm long; capsules 9–34 mm long	**7**
7(6)	Stipules 1.8–2.5 mm long, hispidulous; corolla tubes 5.5–10 mm long, corolla lobes 3–5 mm long; capsules 9–15 mm long	***G.waialealae***
–	Stipules 2–11 mm long, glabrous; corolla tubes 10–28 mm long, corolla lobes 7–18 mm long; capsules 15–34 mm long	**8**
8(7)	Stems usually sharply angled or winged; calyx lobes 0.8–2(–2.5) mm wide; capsules 2(3)-valved without keel	***G.hirtellum***
–	Stems terete or obscurely angled; calyx lobes 3–4 mm wide; capsules 2-valved with keel	***G.degeneri***

## ﻿Preliminary conservation assessment. IUCN Red List Category

*Geniostomaimadae* falls into the Vulnerable (VU) category according to the IUCN criteria VU B1ab(iii)+2ab(iii) which reflects an EOO of 44 km^2^, an AOO of 12 km^2^, only seven small sub-populations consisting of 800–1250 mature plants and a continued decline in quality of habitat inferred. The continued decline in quality of habitat for *G.imadae* is evidenced by severe habitat degradation from invasive non-native mammals such as goats (*Caprahircus* L.), pigs (*Susscrofa* L.), and rats (*Rattus* spp.), along with introduced slugs, insects, and disease. Other serious threats include hurricane force winds, flash floods, landslides triggered after torrential rains, and invasive non-native plants that displace naturally occurring ones within *G.imadae* habitat, especially *Sphaeropteriscooperi* (Hook. ex F. Muell.) R.M.Tryon (Cyatheaceae); *Miconiacrenata* (Vahl.) Michelang. (Melastomataceae); *Psidiumcattleyanum* Sabine (Myrtaceae); *Axonopusfissifolius* (Raddi) Kuhlm. (Poaceae); *Rubusrosifolius* Sm. (Rosaceae); and *Buddlejaasiatica* Lour. (Scrophulariaceae).

Seeds of *Geniostomaimadae* have recently been collected by National Tropical Botanical Garden (NTBG) Science staff and attempts to cultivate plants are being made at both the Hawaii State Division of Forestry and Wildlife (DOFAW) nursery and the NTBG Horticultural Center, Kaua‘i, Hawai‘i.

## Supplementary Material

XML Treatment for Geniostoma imadae

## ﻿Additional information

### Author ORCIDs

Kenneth R. Wood https://orcid.org/0000-0001-6446-1154

David H. Lorence https://orcid.org/0000-0002-6735-9531

Warren L. Wagner https://orcid.org/0000-0001-5012-8422

Susan Fawcett https://orcid.org/0000-0001-5021-1779

### Data availability

All of the data that support the findings of this study are available in the main text.
